# Predictors of hope and despair: Resilience, coping indicators, and demographic characteristics – A longitudinal study

**DOI:** 10.1016/j.ijchp.2026.100679

**Published:** 2026-04-01

**Authors:** Hadas Marciano, Shaul Kimhi, Yohanan Eshel, Bruria Adini

**Affiliations:** a Stress and Resilience Research Center, Tel-Hai - University of Kiryat Shmona in the Galilee, Israel; b The Institute of Information Processing and Decision Making, University of Haifa, Israel; c ResWell Research Center, Tel Aviv University, Israel; d Emeritus at Tel Hai – Tel-Hai - University of Kiryat Shmona in the Galilee, Israel; e Psychology department, University of Haifa, Israel; f Department of Emergency and Disaster Management, School of Public Health, Gray Faculty of Medical and Health Sciences, Tel Aviv University, Israel

**Keywords:** Hope and despair, Societal, Community and individual resilience, Distress symptoms, Sense of danger, Perceived threats, War

## Abstract

Hope and despair are often conceptualized as opposing emotional responses following adversity. While traditionally considered as mutually exclusive, contemporary theoretical frameworks and qualitative research suggest that these emotions may coexist or fluctuate over time. This study examines the relationship between hope and despair in the context of protracted war, exploring their connection with three types of resilience, negative coping factors such as distress, sense of danger, and perceived threats, as well as demographic variables, using quantitative methods. The study is a correlational research based on an internet panel sample of 745 Hebrew-speaking adults in Israel, surveyed at two time points during the ongoing war between Israel and Gaza, with data collected in December 2024 and June 2025. A range of established scales was used to assess the various variables. The findings showed that hope and despair predicted each other only in the first measurement, and, contrary to the assumption that they are opposites, both emotions increased simultaneously from the first to the second measurement. Furthermore, they were predicted by different factors: resilience, particularly societal, individual, and community resilience, predicted hope, while negative coping factors, such as distress symptoms, sense of danger, and perceived threats, predicted despair. These findings suggest that hope and despair are not merely opposites, but rather distinct emotions influenced by different underlying factors. The results underscore the need for further research on the interplay between these emotions and highlight the importance of understanding the mechanisms influencing them in extreme contexts to inform therapeutic and resilience-building interventions.

## Introduction

Hope and despair are often viewed as opposite emotional responses that arise in response to adversity or disaster. For example, Lazarus (1999) described the dichotomous nature of hope and despair in a general context, while Halperin (2016) affirmed this view within the context of war and peace, making it particularly relevant to the present study. According to this point of view, these two emotions embody contrasting states and emotional experiences, as highlighted by Shanahan et al. (2019), who note that the word 'despair' is derived from the Latin term ‘desperare’, which means “down from hope”. Hope is usually associated with positive expectations and the anticipation of better outcomes in the future. For example, Lazarus (1999) claimed that “to hope is to believe that something positive, which does not presently apply to one's life, could still materialize, and so we yearn for it” (Lazarus, 1999, p. 653). In contrast, despair is marked by loss of the very ground of hope and the perception that goals are unattainable (e.g., Steinbock, 2007). The contrasting nature of these two emotions was succinctly conceptualized by Lazarus (1999), who concluded that “we hope because without hope we must despair” (p. 674).

However, numerous qualitative studies across diverse contexts - including palliative care (Olsman et al., 2015), chronic pain (Eaves et al., 2016), HIV/AIDS (Kylmä et al., 2001), Afghan refugees’ migration journeys (Kiriscioglu & Van Heelsum, 2025), and climate activism in New Zealand (Nairn, 2019) - indicate that hope and despair are experienced as dynamic, interwoven emotions that may alternate or coexist rather than function as mere opposites. The current study examines the two fundamental emotions of hope and despair in the context of coping with prolonged war, employing quantitative methods rather than qualitative ones.

Hope is a complex, multifaceted construct that has been extensively studied. According to Snyder (2002), hope entails both the perceived capacity to generate pathways toward desired goals and the self-motivational drive, rooted in agency thinking, to pursue those pathways. Research has shown that hope is associated with positive outcomes, including resilience, well-being, and posttraumatic growth. Specifically, these associations have been observed in extreme contexts, including natural disasters such as Hurricane Harvey (D'Souza et al., 2023; Long et al., 2020), Hurricane Katrina (Tekin et al., 2023), and the 2023 earthquake in Turkey (İme, 2025). Similar patterns have also been documented during man-made disasters, such as among refugees of the Syrian war (Yıldırım et al., 2024), Gazan citizens during the ongoing current Gaza-Israel war (Fekih-Romdhane et al., 2025), and Israeli Jews and Arabs affected by that same conflict (Halperin et al., 2025). Due to the consistent role of hope in elevating psychological well-being and other positive outcomes, it is suggested that hope can serve as a therapeutic target (Cheavens & Whitted, 2023).

Steinbock (2007) identified four challenges to hope - desperation, pessimism, hopelessness, and despair - arguing that despair is the most profound, as it is paradoxically grounded in hope. Unlike hopelessness, which is typically tied to a specific event and leaves the underlying capacity for hope intact, despair confronts the very foundation of hope, simultaneously affirming and rejecting it. This inherent contradiction defines despair’s unique structure: it is more than the absence of hope, revealing hope even in its negation. Thus, while hope accompanies despair, it does not depend on it. We suggest that this view may explain the above-mentioned findings that hope and despair emerge as intertwined emotions that may shift or coexist, rather than existing solely as opposites (Eaves et al., 2016; Kiriscioglu & Van Heelsum, 2024; Kylmä et al., 2001; Nairn, 2019; Olsman et al., 2015). In clinical psychology, the term ‘deaths of despair’ has been proposed to encompass mortality cases linked to suicide, overdoses, and alcohol use (Graham, 2023; Rehder et al., 2021). These authors argue that recognizing the interplay between hope and despair is crucial, as such understanding can inform therapeutic practices and guide collective efforts aimed at fostering resilience and facilitating positive change. The current study seeks to contribute to this endeavor by examining the dynamics between hope and despair in the context of protracted war.

A central concept related to studies of hope and despair is resilience. Resilience refers to the capacity of individuals, groups, or entire societies to effectively cope with threatening events, whether arising from natural disasters or human-made crises (Moya & Goenechea, 2022). Despite its widespread use, there is no universally accepted definition for the term resilience; therefore, its meaning often varies depending on the context in which it is applied (Denckla et al., 2020; Hiebel et al., 2021). Nevertheless, one widely shared view is that resilience refers to the ability to recover from adversity as quickly as possible and to return to normal functioning once the threat has passed or diminished (Bonanno et al., 2015). In this sense, resilience is commonly regarded as an indicator of an individual’s or society’s capacity to cope successfully with stressful situations (Kaim et al., 2024).

In social sciences, it is common to refer to three types of resilience: a) *Societal resilience* (*SR*, also known as national resilience) indicates the ability of a society (a country or nation) to cope and recover from various adversities, such as the Covid-19 pandemic crisis or armed conflicts (Kimhi et al., 2022). b) *Community resilience (CR)* refers to the ability of a local community to recover from a threatening event and return to daily life as quickly as possible (Kimhi et al., 2020). c) *Individual resilience (IR)* refers to the ability of a person (individual) to successfully cope with any crisis or disaster, recover, and return to a level of function similar to the pre-crisis time as quickly as possible or even to 'bounce forward' (Miller-Graff, 2022). Research on individual resilience has shown that changes in a person's function or distress symptoms may still be observed up to two years after a disaster (Kuntz, 2020). A study on hope and resilience has shown that SR, as well as IR, are positively predicted by hope (Marciano et al., 2022). In the current study, we examined the role of three types of resilience - societal, community, and individual resilience - as predictors of both hope and despair.

Additionally, negative coping indicators may affect both resilience and the emotions of hope and despair. These indicators encompass factors where higher levels are associated with poorer coping outcomes at the individual or group level. In the current study, we included three such indicators as predictors of hope and despair: a) *Distress symptoms*, which include various psychological and physical manifestations of depression and anxiety, significantly diminish individuals' quality of life (e.g., Zenger et al., 2010). This negative impact extends beyond psychological well-being, as reflected in the higher mortality risk among individuals with high distress. For example, a meta-analysis confirmed this argument, revealing a 29% increased mortality risk in individuals with high levels of distress compared to those with low levels of distress (Barry et al., 2020). Distress symptoms, particularly anxiety and depressive symptoms, are common and often accompany various adversities, such as the Covid-19 pandemic (e.g., Cooke et al., 2020), terror attacks (e.g., Salguero et al., 2011), and war (e.g., Ahmed et al., 2024; Peleg & Gendelman, 2025). b) *Sense of danger* involves detecting and responding to potential situational and environmental pressures. More specifically, in the context of security and war, this concept refers to individuals' perceptions of fear, worry, and concerns about their own safety and welfare, as well as that of their families and country (Kaniasty et al., 2024). c) *Perceived threats* are conceptualized as anticipations of harm that emerge from cognitive appraisals of events or cues, and are expressed through emotional responses as part of the stress process (Carpenter, 2005). In the context of war, both perceived threats and the sense of danger are central, as they are closely linked to the powerful emotion of fear. As Jarymowicz and Bar-Tal (2006) note, in situations of intractable conflict, fear is associated with concrete dangers, including the risk of death, injury, loss of property, displacement, or severe economic hardship. Such fear is readily aroused and rapidly spread within collectives, often in an automatic manner.

Apart from the resilience and negative coping indicators, several demographic characteristics have been identified in the literature as correlates of hope and/or despair, (as well as more broadly of depressive symptoms and negative coping indicators), although not specifically in the context of war, which is the focus of the current study. Nevertheless, these associations may inform the assumptions underlying the present research. One key variable is religiosity, which is often positively associated with hope (e.g., Sharif et al., 2021), and negatively associated with depressive symptoms and other maladaptive coping indicators (e.g., Coelho-Júnior et al., 2022). Findings regarding age and hope are limited and mixed. Staats (1987) reported no association between age and hope, emphasizing that hope does not decline with age. In contrast, Duggleby et al. (2013) found that among newly diagnosed cancer patients, hope was negatively related to age. However, a recent meta-analysis (Counted et al., 2025) suggests a gradual positive association, with hope increasing across the lifespan. With respect to negative affect, Staats (1987) reported a decline with age, whereas more recent evidence points to a curvilinear (hump-shaped) relationship between age and depression, peaking in midlife (Xu et al., 2024). The relationship between gender and hope is similarly inconsistent. For example, Duggleby et al. (2013) found that men reported higher levels of hope than women among cancer patients, whereas Bjørnnes et al. (2018) found no overall gender differences in hope among cardiac surgery patients, although women scored lower on specific items. In contrast, gender differences are more consistent for despair-related outcomes, with women generally reporting higher levels of distress and maladaptive coping (e.g., Sun et al., 2017). Socioeconomic factors show more robust associations. Both education and income are generally positively related to hope (e.g., Mahendran et al., 2016), whereas lower levels of education and income are consistently linked to higher levels of despair and negative coping indicators across multiple large-scale studies (e.g., Hinata et al., 2021; Park, 2024).

Building on these findings, the present study aimed to examine hope and despair in the context of protracted war, exploring their relationship with three types of resilience (SR, CR, and IR), negative coping factors (distress symptoms, sense of danger, and perceived threats), and various demographic variables. The relationships between these variables were assessed at two time points during the prolonged war with Gaza: fourteen months after its eruption (December 2024) and six months later during the twelve-day war between Israel and Iran (June 2025). We assumed that hope and despair may coexist and be shaped in different directions by individuals’ perceptions and interpretations of war events. Accordingly, we expected to observe changes in these emotions, as well as in their predictors, across the two measurement points.

Specifically, five hypotheses were formulated based on the literature review and previous studies: 1) Hope and despair will be negatively correlated in both measurements. 2) Hope and despair are not simply opposite constructs; thus, they may fluctuate differently over time. Various patterns are possible: both may increase or decrease, either in parallel or independently. 3) Three resilience indicators - SR, CR, and IR - are expected to predict higher levels of hope: the greater the resilience, the higher the reported hope. Conversely, these resilience indicators are also expected to predict lower levels of despair: the higher the resilience, the lower the reported despair. 4) The three negative coping indicators - distress symptoms, sense of danger, and perceived threats - are expected to negatively predict hope: the higher the levels of negative coping, the lower the reported hope, and vice versa. These same indicators are also expected to positively predict despair: the higher the levels of negative coping, the higher the reported despair, and vice versa. 5) If our interpretation that hope and despair are not merely opposites holds, we would expect distinct patterns of predictability to emerge for both emotions, along with variations influenced by the passage of time and other processes related to the events of the war. Finally, regarding other demographic characteristics they will be tested as covariates. Based on prior findings, we assume that religiosity will be positively associated with hope and negatively associated with despair. However, we do not advance specific hypotheses regarding other sociodemographic variables. The literature on age and gender is mixed, and although findings for education and income are more consistent, it remains unclear whether these patterns will generalize to the context of prolonged war.

## Method

Study design and participants. The current study is a correlational research based on an internet panel sample representing the Hebrew-speaking adult population in Israel. The sample included 745 participants (sample’s characteristics are presented in Table 1). The study questionnaire was distributed with the aid of an Internet panel company (Sekernet company; https://sekernet.co.il/), which maintains a panel of over 65,000 people, representing all the groups and sectors of the population. All participants provided informed consent to participate in this study. The ethical committee of Tel Aviv University (#0005985-5 from August 22, 2024) approved the study.

**Table 1:** Demographic characteristics of the respondents (*N* = 745).

Variable	Group	Number of Participants	% of Participants	Average (SD)
Age	1. 18-30	87	11.7	50.58 (15.38)
	2. 31-40	136	18.3	
	3. 41-50	155	20.8	
	4. 51-60	138	18.5	
	5. 61-85	229	30.7	
Gender	1. Male	429	57.6	
	2. Female	316	42.4	
Religiosity	1. Secular	384	51.5	
	2. Traditional	239	32.1	
	3. Religious	78	10.5	
	4. Very religious	44	5.9	
Family income	1. Much lower than the average	137	18.4	2.80 (1.21)
	2. Lower than the average	155	20.9	
	3. Average	249	33.4	
	4. Higher than the average	130	17.4	
	5. Much higher than the average	74	9.9	
Education	1. Elementary or less	3	0.4	
	2. High school	137	18.4	
	3. Higher than high school	188	25.2	
	4. Bachelor's degree	263	35.3	
	5. Master's degree and above	154	20.7	

Data were collected at two different time points during the protracted war between Israel and Gaza. The war erupted on October 7, 2023, following the incursion of Hamas militants and Gazan civilians into southern Israel, and has continued for two years since. The first measurement for the present study was conducted between December 16 and 22, 2024, fourteen months into the war and approximately one week after the fall of the Assad regime in Syria. The second measurement was carried out about six months later, from June 13 to 17, 2025, beginning on the first day of the war with Iran, which commenced with an Israeli attack in the early morning of June 13, 2025. During this 12-day period, Israel experienced continuous missile attacks nationwide, compelling residents to remain near shelters (e.g., Schutz, 2025). Missiles that evaded interception caused substantial property damage and civilian casualties, even among those sheltered (e.g, Fabian, 2025; Lehmann, 2025). This wave of data collection concluded prior to the U.S. bombardment of Iran’s nuclear facilities on the morning of June 22, 2025.

Tools

(a) The hope scale (Jarymowicz & Bar-Tal, 2006; Marciano et al., 2022) consisted of five items. The scale ranges from 1 = 'very little hope' to 5 = 'very high hope'. The higher the score, the higher the hope. Examples of scale items: `I will emerge stronger from the current crisis` and 'My family will emerge stronger from the current crisis.' Alpha Cronbach's reliability of this scale was excellent in both measurements (α = .95, and α = .94, for T1 and T2, respectively).
(b) The despair scale (Kimhi et al., 2025) consisted of five items developed, validated, and pilot-tested explicitly for this study. In the first stage of the study, we pilot-tested a six-item questionnaire among a sample of 50 individuals. Based on the results obtained, one item was deleted, leaving five items that demonstrated good reliability and construct validity. The respondents were asked to describe their general feelings in the past month. The scale ranges from 1 = 'very rarely/not at all' to 5 = 'very often'. The higher the score, the higher the despair. The following are the five items of this new tool: a. 'I feel despair about the situation'; b. 'I see little chance for a positive change.'; c. 'I feel that my life has no meaning'; d. 'I feel helpless in the face of the situation'; and e. 'My family feels despair about the situation'. Alpha Cronbach's reliability of this scale was good in both measurements (α = .81, and α = .82, for T1 and T2, respectively).

To examine the dimensional structure of hope and despair, all ten items from the hope and despair scales in the first measurement were entered into a principal components analysis (PCA) with Varimax rotation. Sampling adequacy was excellent (KMO = 0.88; Bartlett’s test: χ²(45) = 6021.43, *p* < .001). Two components with eigenvalues >1 emerged, accounting for 71.6% of the variance. Communalities were high for most items (≥ .52), except one despair item (.24). All hope items loaded strongly on their component, while reverse-scored despair items also loaded on it to a lesser extent. These results indicate that the combined items reflect a broad underlying continuum while maintaining a distinct despair dimension.

(a) Societal Resilience scale (SR; Kimhi & Eshel, 2019). This scale consists of 16 items, ranging from 1 = 'strongly disagree' to agree 6 = 'strongly agree'. The higher the score, the higher the societal resilience. Examples of scale items: 'I believe that the Israeli government will make the right decisions in times of crisis' and 'Israeli society has coped well with crises in the past and will continue to do so in the future'. Alpha Cronbach's reliability of this scale was very good in both measurements (α = .86, and α = .88, for T1 and T2, respectively).
(b) Individual Resilience (IR; Connor & Davidson, 2003) The scale consists of 10 items, ranging from 0 = 'not true at all' to 4 = 'true almost all the time'. For statistical purposes, however, we revised it to a 1–5 scale. The higher the score, the higher the resilience. Example of scale item: 'I am able to adapt when changes occur'. Alpha Cronbach's reliability of this scale was excellent in both measurements (α = .93, for both T1 and T2).
(c) Community Resilience (CR; Leykin et al., 2013). The scale consists of 10 items, ranging from 1 = 'strongly disagree' to 5 = 'strongly agree'. The higher the score, the higher the resilience. Examples of scale items: 'The municipal authority (municipality/council/secretariat) in my locality is functioning properly' and 'Relations between the different groups in my community are good'. Alpha Cronbach's reliability of this scale was excellent in both measurements (α = .94, and α = .95, for T1 and T2, respectively).
(d) Distress Symptoms Scale (Anxiety and Depression; BSI, Derogatis & Savitz, 2000). The scale consists of five items ranging from 1 = 'not at all' to 5 = 'to a great extent'. The higher the score, the higher the distress. Participants were asked to report how much they had suffered from each symptom during the past month. Examples of scale items: 'Feeling irritable' and 'lack of interest in things’. Alpha Cronbach's reliability of this scale was excellent in both measurements (α = .93, and α = .92, for T1 and T2, respectively).
(e) Sense of Danger (Solomon & Prager, 1992). This scale consists of five items, ranging from 1 = 'not at all' to 5 = 'to a great extent'. The higher the score, the higher the sense of danger. Examples of scale items: 'To what extent do you feel that your life is in danger?' and 'To what extent do you feel that the State of Israel is in existential danger?'. Alpha Cronbach's reliability of this scale was good in both measurements (α = .80, and α = .86, for T1 and T2, respectively).
(f) Perceived Threats. This scale consists of five items. The participants were asked to indicate how much each of the following issues threatened them: security, economic, health, political situation, and violence in Israeli society. The scale ranges from 1 = 'not threatening at all' to 5 = 'very threatening'. The higher the score, the higher the perceived threats. Examples of scale item: 'In the current situation, how would you rate each of the following situations as personally threatening to you?: the security situation'. Alpha Cronbach's reliability of this scale was good in both measurements (α = .79, and α = .81, for T1 and T2, respectively).
(g) demographic characteristics. The following characteristics were examined: personally affected by the war (the question was phrased 'were you or a family member personally affected?'; the answers were 1 = 'no' 2 = 'yes'), level of religiosity ('How would you define yourself religiously? '; 1 = 'secular', 2 = 'traditional', 3 = 'religious', 4 = 'very religious'), level of education ('What is your educational background?' 1 = 'elementary', 2 = 'High school', 3 = 'Non-academic higher than high school', 4 = 'Bachelor's degree', 5 = 'Master's degree and above'), income level ('The average gross income for a family in Israel is currently 21,063 NIS per month. What is your family's income? ' 1 = 'much below the average' to 5 = 'much above the average'), gender (1 = 'male', 2 = 'female'), and age.

## Results

To test Hypothesis 1, which posits that hope and despair will be negatively correlated in both measurements, and to examine whether linear correlations exist between the psychological variables, as required for subsequent regression analyses, we calculated R-Pearson correlations between all eight psychological variables (Table 2, Table 3, for the first and second measurements, respectively). Results indicated the following: a) as expected in hypothesis 1, hope and despair were significantly and negatively correlated in both measurements (r=-.528, *p* < .001 and r=-.379, *p* < .001, for the first and second measurements, respectively). b) All correlations between the psychological variables were significant at both time points (*p* < .001), indicating that the required condition for regression analyses has been met.

**Table 2:** R-Pearson correlations matrix between the psychological variables at the first measurement (December 16-22, 2024; *N* = 745).

	2	3	4	5	6	7	8
1 Despair	-.528⁎⁎	-.410⁎⁎	-.397⁎⁎	-.282⁎⁎	.678⁎⁎	.560⁎⁎	.520⁎⁎
2 Hope	–	.556⁎⁎	.514⁎⁎	.424⁎⁎	-.443⁎⁎	-.369⁎⁎	-.304⁎⁎
3 Societal resilience		–	.305⁎⁎	.487⁎⁎	-.339⁎⁎	-.308⁎⁎	-.331⁎⁎
4 Individual resilience			–	.421⁎⁎	-.488⁎⁎	-.276⁎⁎	-.281⁎⁎
5 Community resilience				–	-.348⁎⁎	-.250⁎⁎	-.261⁎⁎
6 Distress symptoms					–	.499⁎⁎	.433⁎⁎
7 Sense of danger						–	.624⁎⁎
8 Perceived threats							–

⁎⁎ p < .01

**Table 3:** R-Pearson correlations matrix between the psychological variables at the second measurement (June 13-17, 2025; *N* = 745).

	2	3	4	5	6	7	8
1 Despair	-.379⁎⁎	-.479⁎⁎	-.371⁎⁎	-.375⁎⁎	.748⁎⁎	.610⁎⁎	.559⁎⁎
2 Hope	–	.489⁎⁎	.490⁎⁎	.367⁎⁎	-.337⁎⁎	-.277⁎⁎	-.270⁎⁎
3 Societal resilience		–	.297⁎⁎	.515⁎⁎	-.372⁎⁎	-.345⁎⁎	-.349⁎⁎
4 Individual resilience			–	.333⁎⁎	-.459⁎⁎	-.245⁎⁎	-.229⁎⁎
5 Community resilience				–	-.332⁎⁎	-.231⁎⁎	-.218⁎⁎
6 Distress symptoms					–	.589⁎⁎	.501⁎⁎
7 Sense of danger						–	.666⁎⁎
8 Perceived threats							–

⁎⁎ p < .01

**Fig. 1:**
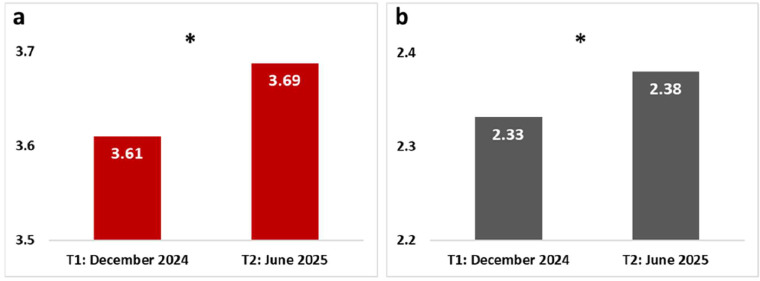
GLM repeated measure of: a. hope and b. despair between the two measurement time points. * - p < .05.

To test Hypothesis 2, which posits that hope and despair may fluctuate differently over time, two repeated-measures general linear models (GLMs) were conducted, with measurement time as the repeated variable, to assess the mean scores of hope and despair, independently. Both analyses were significant, with medium effect sizes {[F(1, 744) = 6.110, p = .014, η²p = .008] and [F(1, 744) = 4.165, p = .042, η²p = .006], for hope and despair, respectively}. Fig. 1 presents the data from these two analyses, showing a significant increase in both hope (Fig. 1a) and despair (Fig. 1b) from the first to the second measurement.

To test Hypotheses 3-5, which concern the predictability of various psychological variables regarding hope and despair, as well as the independence of these two variables, we conducted four two-step regression model analyses. The aim was to examine the unique contributions of the main predictors to the two dependent variables - hope and despair - separately for each of the two measurements. In the first step, demographic covariates (age, gender, education, religiosity level, being personally hurt by the war events, and family income) were entered to control for their potential confounding effects. In the second step, the primary independent psychological variables [SR, CR, IR, distress symptoms, sense of danger, and perceived threats, as well as hope (when the dependent variable was despair), and despair (when it was hope)] were added to the model. This stepwise entry of variables, guided by theoretical and empirical considerations rather than by statistical criteria alone, enabled to evaluate the incremental variance explained at each stage of the model.

Table 4, Table 5 present the results of the two-step regression analyses predicting despair and hope, respectively, in the first measurement. Table 6, Table 7 present the results of the two-step regression analyses predicting despair and hope, respectively, in the second measurement. As observed, both commonalities and differences emerge in the predictability of hope and despair across the two measurements. First, in all four analyses, the inclusion of both psychological and demographic variables in the second step significantly increased the explained variance of the models. Specifically, the explained variance for despair increased from 12.5% to 59.7% and from 12.3% to 64.7% in the first and second measurements, respectively. For hope, the explained variance increased from 7.8% to 51.9% and from 5.6% to 40.1% in the first and second measurements, respectively. Additionally, most of the significant demographic covariates from the first step either became non-significant or remained significant but with reduced effect sizes in the second step of all four analyses. The exception was age in the regression for hope in both measurements, which was not significant in the first step but became significant in the second step with a negative direction, indicating that higher age was associated with lower levels of hope.

**Table 4:** Two-step regression model for mean despair score in the first measurement, predicted by six demographic variables (religiosity, personally hurt, family income, education, age, and gender) and seven psychological variables (hope, SR, IR, CR, distress symptoms, sense of danger, and perceived threats). (December 16-22, 2024; *N* = 745).

Step	Independent variables	Mean Despair score β	p-value
First: Demographic variables only	Religiosity	-.294	p < .001
	Personally hurt	.113	p = .001
	Family income	-.075	p = .044
	Education	.074	p = .044
	Age	-.072	p = .040
	Gender	.177	p < .001
	F	18.680	p < .0001
	R^2^	.125	
Second: Demographic and psychological variables	Religiosity	-.097	p < .001
	Personally hurt	.012	p = .617
	Family income	. 011	p = .663
	Education	-.003	p = .895
	Age	-.012	p = .630
	Gender	.065	p = .009
	Hope	-.194	p < .001
	Societal resilience	-.082	p = .008
	Individual resilience	-.001	p = .962
	Community resilience	.074	p = .011
	Distress symptoms	.432	p < .001
	Sense of danger	.141	p < .001
	Perceived threats	.152	p < .001
	F	85.630	p < .001
	R^2^	.597

**Table 5:** Two-step regression model for mean hope score in the first measurement, predicted by six demographic variables (religiosity, personally hurt, family income, education, age, and gender) and seven psychological variables (despair, SR, IR, CR, distress symptoms, sense of danger, and perceived threats). (December 16-22, 2024; *N* = 745).

Step	Independent variables	Mean Hope score β	p-value
First: Demographic variables only	Religiosity	.254	p < .001
	Personally hurt	-.084	p = .018
	Family income	.047	p = .221
	Education	-.098	p = .009
	Age	. 015	p = .679
	Gender	-.069	p = .059
	F	11.501	p < .001
	R^2^	0.78	
Second: Demographic and psychological variables	Religiosity	.086	p = .002
	Personally hurt	-.033	p = .203
	Family income	-.031	p = .282
	Education	-.030	p = .273
	Age	-.111	p < .001
	Gender	.003	p = .918
	Despair	-.231	p < .001
	Societal resilience	.335	p < .001
	Individual resilience	.299	p < .001
	Community resilience	.084	p = .008
	Distress symptoms	-.006	p = .868
	Sense of danger	-.073	p = .043
	Perceived threats	.103	p = .003
	F	62.826	p < .001
	R^2^	.519

**Table 6:** Two-step regression model for mean despair score in the second measurement, predicted by six demographic variables (religiosity, personally hurt, family income, education, age, and gender) and seven psychological variables (hope, SR, IR, CR, distress symptoms, sense of danger, and perceived threats). (June 13-17, 2025; *N* = 745).

Step	Independent variables	Mean Despair score β	p-value
First: Demographic variables only	Religiosity	-.284	p < .001
	Personally hurt	.097	p = .005
	Family income	-.084	p = .022
	Education	.088	p = .015
	Age	-.126	p < .001
	Gender	.181	p < .001
	F	18.221	p < .001
	R^2^	.122	
Second: Demographic and psychological variables	Religiosity	-.069	p = .004
	Personally hurt	.006	p = .799
	Family income	-.007	p = .779
	Education	.016	p = .499
	Age	.008	p = .722
	Gender	.020	p = .402
	Hope	-.044	p = .125
	Societal resilience	-.140	p < .001
	Individual resilience	-.004	p = .892
	Community resilience	-.034	p = .204
	Distress symptoms	.512	p < .001
	Sense of danger	.134	p < .001
	Perceived threats	.131	p < .001
	F	105.746	p < .001
	R^2^	.647

**Table 7:** Two-step regression model for mean hope score in the second measurement, predicted by six demographic variables (religiosity, personally hurt, family income, education, age, and gender) and seven psychological variables (despair, SR, IR, CR, distress symptoms, sense of danger, and perceived threats). (June 13-17, 2025; *N* = 745).

Step	Independent variables	Mean Hope score β	p-value
First: Demographic variables only	Religiosity	.224	p = .001
	Personally hurt	-.019	p = .594
	Family income	.046	p = .228
	Education	-.086	p = .022
	Age	.021	p = .560
	Gender	-.082	p = .025
	F	8.365	p < .001
	R^2^	.056	
Second: Demographic and psychological variables	Religiosity	.107	p < .001
	Personally hurt	.012	p = .682
	Family income	-.027	p = .390
	Education	-.048	p = .112
	Age	-.097	p = .001
	Gender	.022	p = .470
	Despair	-.074	p = .125
	Societal resilience	.302	p < .001
	Individual resilience	.387	p < .001
	Community resilience	.072	p = .037
	Distress symptoms	.033	p = .479
	Sense of danger	-.069	p = .109
	Perceived threats	.078	p = .052
	F	33.337	p < .001
	R^2^	.401	

Examining the second step of the four analyses, which incorporated all 13 predictive variables, the following results were observed: a) Despair significantly and negatively predicted hope, with higher levels of despair associated with lower hope, and hope significantly and negatively predicted despair, with higher hope associated with lower despair. However, this pattern was observed only in the first measurement. In the second measurement, neither variable predicted the other. b) As predicted by the first part of Hypothesis 3, the three types of resilience - SR, CR, and IR - positively and significantly predicted hope in both measurements, with higher resilience associated with higher hope. However, regarding despair and contrary to the second part of Hypothesis 3, SR negatively and significantly predicted despair only in the second measurement, with lower SR associated with higher despair, and only CR significantly predicted despair in the first measurement, but indicated a non-intuitive direction that higher CR was associated with higher despair. c) As predicted by the second part of Hypothesis 4, distress symptoms, sense of danger, and perceived threats were all positive and significant predictors of despair in both measurements, with higher scores associated with higher despair. However, in contrast with the first part of Hypothesis 4, only perceived threats significantly (in the first measurement) or marginally (in the second measurement) predicted hope, but showed a non-intuitive direction, where higher perceived threats were associated with higher hope. In contrast, sense of danger was a significant negative predictor of hope in the first measurement, indicating, in line with our hypothesis, that higher perceived danger was associated with lower levels of hope. d) In line with our hypothesis, religiosity negatively and significantly predicted despair and positively and significantly predicted hope in both measurements, with higher religiosity associated with lower levels of despair and higher levels of hope. Gender significantly predicted despair only in the first measurement, with being female associated with higher levels of despair. Age negatively and significantly predicted hope in both measurements, with older age associated with lower levels of hope. No other demographic variables were found to be associated with despair or hope in the second step of the regression analyses.

## Discussion

The study examined the association between hope and despair at two time points during the protracted war that began with a traumatic surprise attack by Hamas on communities in Israel – fourteen months after the war erupted and six months later, during the 12-day conflict with Iran. Specifically, we investigated how hope and despair fluctuated over time with events of the war, how they are predicted by three types of resilience (SR, IR, and CR) and three negative coping indicators (distress symptoms, sense of danger, and perceived threats), and whether demographic characteristics, including age, gender, religiosity level, being personally hurt by the war events, family income, and education level, served as covariates to predict these emotions differently.

The results mainly supported our hypotheses confirming that hope and despair can coexist. First, although the association between hope and despair was negative and significant in both measurements, it was only of medium size, supporting the claim that hope and despair are not necessarily opposite (Steinbock, 2007). Additionally, the fluctuations over time indicated that both hope and despair significantly increased in the second measurement compared to the first. This pattern would not be observed if the two emotions were merely opposites, as one would expect that when one emotion increases, the other would decrease. This trade-off relationship is commonly expected by individuals with a spiritual perspective (e.g., Ejiogu, 2025). Furthermore, when examining the second step of the regression analyses, it was found that despair significantly and negatively predicted hope, and hope significantly and negatively predicted despair, only in the first measurement. In contrast, in the second measurement, neither variable predicted the other.

Together, these patterns of results support our assumption of the independence of hope and despair. The medium-sized correlation, the simultaneous increase of both emotions, and their lack of consistent predictive relationship suggest that hope and despair are distinct concepts or emotions, shaped by different processes and events. These results diverge from several earlier theoretical conceptions suggesting that hope and despair are opposing emotions (Govier, 2011; Halperin, 2016; Lazarus, 1999). In contrast, our findings align with the view of other theorists that hope and despair, while antonyms representing opposing emotional poles, are not entirely dichotomous and may coexist (Kwong, 2024; Meirav, 2009; Steinbock, 2007).

Second, the regression analyses revealed that including the psychological variables in the second step (beyond the demographic variables in the first step) significantly and highly increased the explained variance in all models. Additionally, most of the significant demographic covariates from the first step either became non-significant or remained significant but with reduced effect sizes in the second step across all four analyses. The only exception was age, which emerged as a significant negative predictor of hope in both measurements. These findings suggest that psychological variables have a stronger influence on predicting hope and despair, while demographic factors play a less central role in these predictions. This conclusion aligns with large-scale international research that also reported the pivotal role of psychological factors over demographic variables in predicting hope for the future (McGrath, 2023).

Third, the three types of resilience variables - SR, IR, and CR - positively and significantly predicted hope in both measurements. However, only SR negatively and significantly predicted despair, and only in the second measurement, while only CR significantly predicted despair in the first measurement, with a counterintuitive direction (higher CR associated with higher despair). In contrast, all three negative coping variables - distress symptoms, sense of danger, and perceived threats - positively and significantly predicted despair in both measurements. However, only sense of danger negatively and significantly predicted hope in the first measurement, while perceived threats positively and significantly (or marginally significantly) predicted hope in both measurements, with a counterintuitive direction, as higher perceived threats were associated with higher hope. These findings suggest that hope and despair are influenced by different variables. While hope was found to be mainly and consistently predicted by the positive resilience variables (the higher the resilience the higher the hope), despair was consistently predicted by negative coping mechanisms of distress symptoms, sense of danger, and perceived threats (the higher these factors the higher the despair). The findings align with a previous meta-analysis of 77 studies, which revealed that hope is strongly predicted by positive variables such as life satisfaction and social support, moderately predicted by depression, and only weakly predicted by other negative variables, such as stress (Yarcheski & Mahon, 2016).

Furthermore, the findings further emphasize the independence of the two emotions and also suggest mechanisms that may underlie this independence. While hope is more influenced by positive factors that enhance societal, community, and individual resilience, despair is more affected by negative factors that increase distress, sense of danger, and perceived threats. Given that both negative and positive processes and events can occur during difficult times, both despair and hope may be influenced simultaneously but independently.

The pattern of results, showing that both hope and despair increased from the first to the second measurement, that they were not always significant predictors of one another, and that each was primarily associated with different psychological variables, supports our contention that these emotions are not simple opposites. If despair was merely the negation of hope, we would expect an inverse relationship such that decreases in hope correspond with increases in despair, that each emotion would consistently predict the other, and that they would share the same predictors but in opposing directions. Our findings clearly indicate that this is not the case.

From a more applied perspective, our results suggest that the relationship between hope and despair is complex and intertwined rather than strictly polarized. This phenomenon has been noted in previous qualitative studies (Eaves et al., 2016; Kiriscioglu & Van Heelsum, 2024; Kylmä et al., 2001; Nairn, 2019; Olsman et al., 2015). However, given the scarcity of research employing quantitative methods on this topic, further empirical investigation is warranted to substantiate this claim.

Two unexpected findings emerged in the current study, both of which contradict our initial hypotheses and merit further discussion: a) The positive relationship between perceived threats and hope across both measurements. Although counterintuitive, the positive association between perceived threats and hope was also reported in previous findings showing a moderating effect of hope on the perception of threats in the context of the Russia–Ukraine war (Slezáčková et al., 2024). Similarly, Dean and Wilson (2023) found a positive relationship between perceived threats and hope in the context of climate change and conservation, and suggested that hope pathways are mediated by stronger threat appraisals, indicating that hopeful individuals may actually perceive threats more accurately while maintaining confidence in their ability to act. Nevertheless, this effect may also relate to the concept of optimism bias, the tendency to fail to incorporate bad news into prior beliefs, leading to positively biased views (Garrett et al., 2018). Although they found that the bias disappears under high acute perceived threat, the authors noted that prolonged threat could enhance the integration of negative information over time, potentially causing psychiatric problems. Therefore, we suggest that individuals experiencing high, prolonged perceived threats may need hope to cope with the crisis in order to prevent such harmful consequences. This process may explain the association between hope and perceived threats observed in the current study. Further research is needed to differentiate between these two explanations. b) The positive relationship between community resilience (CR) and despair at the first measurement. Although community resilience is typically regarded as a protective factor (e.g., Abate et al., 2024), negative outcomes associated with community resilience have also been reported. For example, Chaigneau et al. (2022) explored the relationship between resilience and well-being, providing examples from various contexts where prioritizing one compromised the other. Similarly, Mguni et al. (2012) identified all possible associations between well-being and resilience in a large-scale study in England. Some communities exhibited high levels of both well-being and resilience, while others showed low levels of both. Additionally, some communities had high well-being but low resilience, while others, resembling our findings concerning hope and CR, were characterized by low well-being but high resilience. Regarding our findings, one possible explanation lies in the context of a war that began following an extreme unprecedented adversity, characterized by extensive casualties, mortality, and subsequent economic crises. Under such extreme conditions, community resilience may be associated with heightened strains and tensions within the community (e.g., George & Stark, 2016). The observed positive correlation between community resilience and despair warrants further investigation across diverse communities severely affected by conflict.

Limitations. The present study requires consideration of its limitations. First, a key limitation of this study is its focus on a specific time frame and sociopolitical context, which may constrain generalizability. However, a notable strength is that the entire sample was exposed to the same unfolding events at the same time, an advantage rarely achievable in studies of individual-level crises. This shared context allows for a more controlled examination of the dynamic interplay between hope and despair, while still offering insights into broader psychological responses to adversity. Second, it is worth noting that this is a correlational study, which does not permit causal inferences; however, its longitudinal design helps to mitigate this limitation. Third, based on an internet panel, the current sample cannot guarantee the representativeness of Israeli society, mainly because it does not include Arabic speakers (due to technical constraints).

Conclusions. The current study demonstrates that hope and despair are complex emotional states that, rather than being mere polarized opposites as many believed, often coexist and intertwine in the aftermath of adversity. Our findings support the view that hope and despair are not inherently opposed, and that the factors influencing each may differ. Given that these emotions play a pivotal role in coping with challenges, that despair is largely viewed as a source of significant psychological distress and even a cause of mortality, and that a common therapeutic goal is to enhance individuals’ hope, it is important to recognize that increasing hope does not necessarily reduce despair, as evidenced in the present study. Understanding the nuances between these two emotions, as well as the factors that uniquely affect each, is crucial for better comprehending how adversity may shape emotional responses. This understanding has significant implications for interventions aimed at fostering hope while simultaneously alleviating despair.

## Declaration of competing interest

The authors declare that they have no known competing financial interests or personal relationships that could have appeared to influence the work reported in this paper.
